# Comparison of Body Mass Index, Energy and Macronutrient Intake, and Dietary Inflammatory Index Between Type 2 Diabetic and Healthy Individuals

**DOI:** 10.34172/jrhs.2025.174

**Published:** 2024-12-25

**Authors:** Nazanin Cheloi, Zeynab Asgari, Solale Ershadi, Rozita Naseri, Amrollah Sharifi

**Affiliations:** ^1^Department of Nutrition and Food Hygiene, School of Medicine, Hamadan University of Medical Sciences, Hamadan, Iran; ^2^Student Research Committee, Hamadan University of Medical Sciences, Hamadan, Iran; ^3^Department of Internal Medicine, Kermanshah University of Medical Sciences, Kermanshah, Iran; ^4^Nutrition Health Research Center, Hamadan University of Medical Sciences, Hamadan, Iran

**Keywords:** Diabetes mellitus, Nutrition assessment, Diet, Body mass index, Dietary inflammatory index

## Abstract

**Background:** Type 2 diabetes mellitus (T2DM) is a chronic disorder diagnosed by elevated blood sugar. Key risk factors for T2DM include obesity, a sedentary lifestyle, and poor dietary habits. The proportion of macronutrients and the dietary inflammatory index (DII) seem to be associated with the risk of T2DM. This study aimed to assess and compare the macronutrient intake, DII, and BMI of newly diagnosed T2DM patients with healthy individuals in Kermanshah, Iran.

**Study Design:** This study employed a case-control design.

**Methods:** A total of 105 newly diagnosed T2DM patients were selected as the case group, while an equal number of control participants were selected from their non-diabetic friends or neighbors. Dietary intake was assessed using a validated food frequency questionnaire. Energy, macronutrients, fatty acids intake, and DII were estimated using ShaFA software. Statistical significance was set at *P* values below 0.05.

**Results:** The study included 105 newly diagnosed T2DM and 105 healthy individuals. Diabetic patients had significantly lower intake of protein, total fat, polyunsaturated fatty acids (PUFA), and monounsaturated fatty acids (MUFA), while their body mass index (BMI) and DII were higher. Multiple logistic regression indicated that protein, PUFA, and MUFA are protective factors for T2DM, while BMI, carbohydrates, and saturated fat intake are risk factors. A higher DII was correlated with an increased risk of T2DM risk, even after adjusting for BMI.

**Conclusion:** Lower BMI and DII, balanced macronutrient intake, and consumption of MUFA and omega-3 fatty acids may be beneficial in preventing or delaying the onset of T2DM. Further research is needed to explore these associations in greater depth.

## Background

 Diabetes mellitus (DM) is a chronic metabolic disorder characterized by persistent hyperglycemia. This condition can result from impaired insulin secretion, insulin resistance, or a combination of both.^1^ It is a global public health burden, with the number of patients expected to increase to 200 million by 2040.^1,2^ Type 2 diabetes mellitus (T2DM) is the most prevalent form of DM, accounting for approximately 95% of DM cases in most populations. Risk factors for T2DM include aging, obesity, overeating, dietary habits such as higher intake of animal fats and sugary beverages, sedentary lifestyle, high blood pressure, and hyperlipidemia.^2-4^

 Epidemiological studies have indicated that increased energy intake is associated with higher rates of obesity and metabolic disorders. It has been demonstrated that a high-calorie diet, abundant in glucose and fatty acids, can trigger epigenetic alterations in individuals with diabetes.^5-8^ Moreover, overeating can decline mitochondrial function, leading to an elevated conversion of oxygen to superoxide, which is a pathological process in diabetes.^5^ Nutritional interventions are among the most effective ways to prevent and manage T2DM.^9^ Furthermore, changing the pattern of macronutrient intake, such as reducing carbohydrates and increasing fiber intake, enhanced insulin sensitivity.^6,9-11^

 The dietary inflammatory index (DII), a tool to evaluate the overall inflammatory potential of a diet, has been recognized as an influential factor in many chronic diseases in recent years. The DII is calculated using 45 types of food items. A high DII score indicates the inflammatory potential of the diet, while a low DII score indicates an anti-inflammatory potential.^12^ Various studies have demonstrated that a diet with a high DII score is associated with increased systemic inflammation and the risk of many chronic diseases.^13-15^ Some recent studies have also reported a relationship between DII and T2DM.^16,17^ This study aimed to assess and compare the macronutrient intake, DII, and body mass index (BMI) of newly diagnosed T2DM patients with healthy individuals in Kermanshah, Iran.

## Methods

 In this case-control study, newly or more recently diagnosed T2DM adult patients from private or academic clinics in Kermanshah, Iran, in 2023 were invited to participate as the case group. Volunteers in the control group were selected among friends or neighbors of each patient, with similar gender and relatively similar age ( ± 5 years).

 Considering a type I error probability of 0.05, a type II error probability of 0.8, and a 1:1 ratio for the case and control groups, the sample size of 105 people per group was estimated based on the results of Zaroudi and colleagues’ study.^18^

 The inclusion criteria for the patients were a definitive diagnosis of T2DM within the last three months by a specialist, according to American Diabetes Association criteria,^19^ non-adherence to a special diet or significant changes in the diet during the last three months before the study, and willingness to voluntarily participate in the study. The exclusion criteria included pregnancy and breastfeeding, as well as the presence of other diseases that led to significant diet changes in the last three months. The control group adhered to the same inclusion and exclusion criteria, except for the T2DM diagnosis.

 The gender, age, and anthropometric characteristics (height, weight, and BMI) of the participants were recorded. In addition, the participants completed the 168-item validated semi-quantitative food frequency questionnaire (FFQ) with guidance from trained researchers.^20^ Calorie, carbohydrate, protein, total fat, saturated fatty acids (SFA), monounsaturated fatty acids (MUFA), polyunsaturated fatty acids (PUFA), omega-3 fatty acids, and dietary fiber intake data were estimated using the FFQ data through the ShaFA food analysis software.^21^

 The DII was calculated according to Shivappa and colleagues’ study,^12^ with the modification, that out of 45 food items, 38 items were used for DII calculation, while eugenol, flavan-3-ol, flavones, flavonones, flavonols, anthocyanidins, and isoflavones were excluded due to the lack of dietary intake data. Proper statistical analyses were performed, including logistic regression and independent sample t-test. *P* values lower than 0.05 were considered significant.

 This study was carried out following the approval of the ethics committee of Hamadan University of Medical Sciences (ethic code IR.UMSHA.REC.1401.498, available at https://ethics.research.ac.ir/EthicsProposalView.php?id=280603). Informed written consent was obtained from all volunteers before the study.

## Results

 A total of 105 newly diagnosed T2DM patients (37 men and 68 women) and 105 healthy people (37 men and 68 women) were included in the study. The mean age and mean energy intake, carbohydrate, fiber, and saturated fat intake were not statistically significant between the two groups. However, food intake of protein (*P* < 0.001), total fat (*P* < 0.001), PUFA (*P* < 0.001), and MUFA (*P* < 0.001) were significantly lower in diabetic patients compared to the control group. The diabetic group consumed more carbohydrates than the healthy group, but this difference was not statistically significant. On the other hand, the BMI (*P* < 0.001) and the DII (*P* = 0.005) were significantly higher in diabetic patients than in the control group (Table 1).

**Table 1:** Descriptive statistics and comparisons of age, BMI, DII, energy intake, and the consumption of macronutrients and fatty acids between diabetic patients and healthy control groups

**Variables**	**Case Group**				**Control Group**				***P*** ** value**
	**Mean**	**SD**	**Median**	**IQR**	**Mean**	**SD**	**Median**	**IQR**	
Age (y)									
Men	53.0	13.7	50.0	17.0	53.5	14.0	55.0	16.0	0.782
Women	53.6	10.2	53.0	14.0	52.8	10.4	52.0	16.0	0.654
Total	53.4	11.5	53.0	15.0	53.0	11.6	53.0	15.0	0.830
BMI (kg/m^2^)									
Men	27.11	3.59	26.85	3.80	25.60	2.83	26.25	3.86	0.875
Women	29.88	6.00	28.02	6.32	26.72	3.80	26.73	3.47	0.001
Total	28.90	5.43	27.41	4.94	26.35	3.53	26.54	3.75	0.001
Energy (Calorie)									
Men	2548	747	2624	914	2757	661	2603	862	0.211
Women	2462	717	2538	1137	2559	715	2657	949	0.432
Total	2492	725	2616	1113	2625	700	2603	880	0.179
Carbohydrate (g)									
Men	368	112	384	93	358	95	354	120	0.755
Women	362	129	386	200	333	109	328	162	0.166
Total	364	123	384	192	347	106	343	155	0.234
Protein (g)									
Men	85.1	26.8	87.6	28.6	98.7	22.1	99.9	28.1	0.024
Women	79.1	24.5	79.7	36.2	91.8	29.9	95.7	32.5	0.008
Total	81.2	25.3	84.1	31.2	94.1	27.6	96.3	30.7	0.001
Total fat (g)									
Men	90.9	37.3	85.8	36.1	105.6	32.8	101.2	29.5	0.034
Women	85.5	27.2	84.4	31.7	104.0	32.8	108.1	33.8	0.001
Total	87.4	31.0	85.2	33.5	104.5	32.7	106.1	32.0	0.001
SFA (g)									
Men	30.84	15.71	28.03	17.78	29.66	10.99	29.83	11.86	0.715
Women	29.15	12.86	26.90	18.64	30.00	14.60	30.00	15.60	0.733
Total	29.74	13.88	26.99	18.11	29.85	13.47	29.85	13.65	0.954
PUFA (g)									
Men	19.51	8.84	18.01	10.09	25.61	9.07	24.50	7.76	0.003
Women	18.16	7.16	17.60	9.06	24.06	8.71	24.37	9.20	0.005
Total	18.64	7.78	17.73	9.13	24.58	8.82	24.47	9.09	0.001
MUFA (g)									
Men	31.55	15.40	30.19	20.72	40.69	14.16	39.92	12.82	0.007
Women	29.54	12.85	29.62	16.65	40.79	13.01	40.69	15.70	0.011
Total	30.25	13.76	29.70	17.16	40.76	13.33	40.59	14.44	0.001
Dietary fiber (g)									
Men	35.1	12.7	35.4	16.2	36.4	9.0	38.3	13.1	0.706
Women	33.0	14.0	34.4	24.7	31.7	10.9	31.8	15.8	0.486
Total	33.7	13.5	35.1	20.2	33.2	10.7	33.1	14.1	0.652
DII									
Men	-1.000	2.209	1.372	2.701	-1.852	1.125	-2.300	1.406	0.045
Women	-0.520	2.357	-1.563	4.091	-1.275	1.874	-1.928	2.011	0.039
Total	-0.689	2.307	-1.477	3.344	-1.467	1.679	-2.043	1.898	0.005

*Note.* BMI: Body mass index; DII: Dietary inflammatory index; SD: Standard deviation; IQR: Interquartile range; SFA: Saturated fat; MUFA: Monounsaturated fatty acids; PUFA: Polyunsaturated fatty acids. Significance level: *P* < 0.05.

 In a multiple logistic regression model, with included BMI and dietary intake of protein, carbohydrates, fiber, MUFA, PUFA, and SFA, it was found that protein, PUFA, and MUFA have a protective role, whereas BMI, carbohydrates, and SFA were risk factors for T2DM (Table 2).

**Table 2:** multiple logistic regression model (backward stepwise), including BMI and dietary intake of protein, carbohydrates, fiber, MUFA, PUFA, and SFA

**Variables**	**OR (95% CI)**	***P*** ** Value**
BMI (kg/m^2^)	1.19 (1.07, 1.32)	0.001
Protein (g)	0.94 (0.91, 0.97)	0.001
Carbohydrate (g)	1.01 (1.01, 1.02)	0.005
Fiber (g)	1.07 (1.00, 1.02)	0.092
MUFA (g)	0.94 (0.89, 0.99)	0.030
PUFA (g)	0.89 (0.81, 0.99)	0.033
SFA (g)	1.06 (1.02, 1.10)	0.001
Constant	0.05 (0.06, 1.19)	0.064

*Note.* BMI: Body mass index; OR: Odds ratio; CI: Confidence interval; MUFA: Monounsaturated fatty acids; PUFA: Polyunsaturated fatty acids; SFA: Saturated fat. Significance level: *P* < 0.05.

 Logistic regression analysis showed that a higher DII is associated with an increased risk for T2DM (OR: 1.215; 95% CI: 1.055-1.399; *P* = 0.007), even after adjusting for BMI (OR: 1.212; 95% CI: 1.044- 1.406; *P* = 0.011).

## Discussion

 In this study, the assessment of 105 newly diagnosed T2DM adult patients and their comparison with 105 healthy individuals showed that the diabetic group has a higher BMI and consumed food with a higher DII and lower intakes of protein, total fat, PUFA, and MUFA.

 Our findings, consistent with many other studies,^22-24^ indicated that higher BMI is a risk factor for developing diabetes. Excess energy intake is associated with fat accumulation in the body, which increases BMI and subsequently raises the risk of T2DM.^25^ Experimental studies indicated that an increase in body weight, through complex biological processes, especially fat hypoxia, stimulates adipose tissue fibrogenesis and macrophage chemotaxis. This process can suppress the catabolism of branched-chain amino acids in adipose tissue. In addition, it increases the number and ratio of pro-inflammatory immune cells in adipose tissue, decreases adiponectin secretion production, and increases serum-free fatty acid concentrations, all of which contribute to an increased risk of diabetes.^25,26^

 In a study by Breen et al,^27^ energy intake in diabetic subjects was similar to that in non-diabetic subjects. However, diabetic subjects consumed more monounsaturated fats, polyunsaturated fats, and protein, while their consumption of carbohydrates, non-milk sugar, and fiber was significantly lower. It should be noted that in the study by Breen et al, patients were previously diagnosed, so their diet most likely might be affected by the disease. On the other hand, our study included newly or more recently diagnosed patients, which minimizes the risk of information bias due to intentional therapeutic changes in dietary patterns.

 There is a theory that high-carbohydrate diets enhance weight gain due to their tendency to increase insulin secretion.^28^ To date, various low-carbohydrate diets such as Atkins, Zone, and Ketogenic diets have been promoted for weight loss, as well as the prevention and treatment of diabetes. However, the effectiveness of such diets remains a topic of debate, especially since significant decreases in carbohydrate intake are usually accompanied by increased total fat consumption.^28^ The study by Thanh et al^29^ showed that carbohydrates constitute a large part of energy intake in diabetic patients. Additionally, a cohort study in the United States showed that a high intake of starchy foods and a low intake of dietary fiber were associated with an increased risk of T2DM.^30^ Other studies have also indicated that high carbohydrate intake is linked to a higher risk of developing T2DM,^29^ and it may even be associated with an increased risk of elevated blood sugar levels in affected people.^10,31^ Both the amount and type of carbohydrates affect blood sugar levels; however, the amount of carbohydrates seems to have a more significant effect.^32,33^

 On the other hand, a meta-analysis study suggested that strict carbohydrate restriction does not have a considerable long-term impact on blood sugar control in diabetic patients.^34^ It is theorized that the beneficial effects observed in some studies that prescribed low-carb diets are primarily due to weight loss, rather than reduced carbohydrate intake.^28^ In addition, fiber intake is lower in diets with severe carbohydrate restriction.^28^ Dietary fiber is a protective factor for T2DM,^30^ and sufficient fiber intake significantly lowers blood sugar levels in diabetic patients and is associated with reduced mortality.^31^ In a similar study conducted in Tabriz (Iran), a higher intake of fiber and vegetable proteins was associated with a lower risk of T2DM.^35^ Furthermore, regarding protein intake, a cross-sectional study in Tunisia showed that higher protein intake is associated with improved glycemic control.^36^

 Controversy exists regarding the association between omega-3 fatty acids and the risk for T2DM incidence.^37^ While omega-3 consumption reduces the risk of T2DM in Asian countries, it appears to increase the risk of T2DM in Western Europeans and Americans.^38-42^ However, using biomarkers showed no increased risk of T2DM associated with omega-3 fatty acids in one American and two Finnish cohorts. ^43-45^ The findings in the present study suggested that higher omega-3 intake is protective against the development of T2DM.

 Furthermore, total fat intake was not associated with the risk of developing T2DM.^37^ Moreover, a meta-analysis found that saturated fat intake is not linked to T2DM.^46^ A prospective study in Spain showed that total dietary fat, MUFA, PUFA, and trans fatty acids are not related to the incidence of T2DM.^47^ Additionally, the Nurses’ Health Studies I and II found that women who consumed approximately 8 grams of olive oil per day (a rich source of MUFA) had a 10% lower risk of developing T2DM than those who never consumed olive oil.^48^ However, a prospective study indicated that fasting serum MUFA predicts worsening hyperglycemia and was associated with an increased risk of developing T2DM.^49^

 Nevertheless, two interventional studies support improved insulin sensitivity after MUFA consumption.^50,51^ In our study, the diabetic group consumed lower MUFA than the control group, as well as lower total fat. Since reduced fat and protein intake often leads to higher carbohydrate consumption for equal energy intake, it can be concluded that people who devote a more significant portion of their caloric intake to carbohydrates have higher odds of developing diabetes.

 The findings from the NHANES (2013-2014) study revealed that diabetic patients have a higher DII than healthy controls. It should be noted that the diabetic group was not newly diagnosed.^52^ A similar study in Mexico City found that individuals in the highest DII quintile have higher odds of developing T2DM.^53^ The results of the E3N cohort study also showed that a diet with a higher anti-inflammatory potential is associated with a lower risk of T2DM incidence.^54^ The results of two studies in Iran (Isfahan) and the United States (NHANES 2007-2016) also showed that a pro-inflammatory diet is associated with a high risk of prediabetes.^55^ A recent meta-analysis study found no significant association between DII and the risk for T2DM; however, considerable heterogeneity was observed. Moreover, higher DII was significantly associated with an increased risk of T2D in high-quality studies.^56^ Furthermore, another meta-analysis showed that higher DII was linked to a higher risk of developing T2DM.^57^ Our study similarly found that higher DII (a more pro-inflammatory diet) is associated with higher odds of T2DM incidence.

 In conclusion, lower BMI, lower DII, a balanced intake of macronutrients, and higher consumption of MUFA and omega-3 fatty acids seem beneficial for preventing or postponing the development of T2DM. Further studies are suggested. A limitation of this study was the lack of laboratory examination in the control group to detect hidden DM. In addition, due to the retrospective nature of the FFQ, dietary intake data may be subject to recall bias.

**Highlights**

- Diabetic patients had a significantly lower intake of protein, total fat, PUFA, and MUFA.
- Diabetic patients had a significantly higher BMI and DII.
- Higher intake of protein, PUFA, and MUFA intake were protective for T2DM.
- Higher BMI, carbohydrate intake, and saturated fatty acids were risk factors for T2DM.

## Competing Interests

 None.

## Ethical Approval

 This study was carried out following the approval of the ethics committee of Hamadan University of Medical Sciences (ethic code IR.UMSHA.REC.1401.498).

## Funding

 This study was supported by Hamadan University of Medical Sciences (grant number: 39222).
